# What Makes Community Inclusion Work for Older Adults With an Intellectual Disability? An Application of the Normalisation Process Theory

**DOI:** 10.1111/jar.70317

**Published:** 2026-10-11

**Authors:** L. Taggart, A. Campbell, I. Doherty, K. Ensue, H. Ahmed, A. Hanna, L. Armstrong, B. Bunting, A. Ryan, C. O'Neill, M. Clarke, B. Leahy, S. Willis, J. Schofield, R. Stancliffe, A. Hassiotis

**Affiliations:** ^1^ Queen's University Belfast Belfast UK; ^2^ University College London London UK; ^3^ Volunteer Now Belfast UK; ^4^ Ulster University Derry UK; ^5^ Central and North West London NHS Foundation Trust London UK; ^6^ Compass Advocacy Network Limited Ballymoney, Northern Ireland UK; ^7^ The University of Sydney Sydney Australia

**Keywords:** community groups, intellectual disability, MATILDA intervention, mechanisms, normalisation process theory, social processes

## Abstract

**Background:**

Older adults with intellectual disability experience high levels of loneliness and social exclusion. MATILDA is a community‐based intervention supporting participation in mainstream community groups through volunteer mentors.

**Aim:**

To use Normalisation Process Theory (NPT) to explore the processes, mechanisms, and barriers influencing implementation and sustainability.

**Methods:**

A qualitative study (focus groups and interviews with 29 adults with intellectual disability, volunteer mentors, intellectual disability staff, and community‐group managers) across two UK sites. Data analysed using an NPT‐informed framework.

**Findings:**

Implementation was supported through sense‐making, engagement, collective action, and appraisal. Key mechanisms included Volunteer Co‐ordinators, mentor support, participant‐group matching, adaptable community groups, and strong relationships. Community inclusion emerged as relational work. Staffing pressures, transport difficulties, and resource constraints limited participation for some individuals.

**Conclusions:**

MATILDA shows potential as a sustainable community inclusion intervention. Successful implementation depends on programme components and the relational and organisational work needed to support participation and belonging.

**Trial Registration:**

ISRCTN, ISRCTN15294181. Registered 20th December 2022, https://doi.org/10.1186/ISRCTN15294181

## Lay Summary

- Research has shown that many befriending schemes and community‐based interventions for adults with intellectual disability fail to be effectively implemented and sustained in everyday real‐world settings.
- The MATILDA intervention is a low‐cost, mainstream community‐based intervention that places explicit emphasis on group integration and sustainability.
- Applying the Normalisation Process Theory enables a systematic exploration of how MATILDA was understood, functioned, and evaluated across the different stakeholder groups.
- Findings from this study demonstrated that the MATILDA intervention was acceptable, adaptable, feasible, implementable, and sustainable, with strong potential for embedding the MATILDA intervention across a range of mainstream older people's community‐based groups.
- By leveraging existing community infrastructure and volunteer capacity, MATILDA has the potential to provide a low‐cost, sustainable intervention that reduces loneliness and mental ill‐health for adults with an intellectual disability while decreasing reliance on formal health and social care services.

## 1 Introduction

The concept of community participation for people with an intellectual disability (ID) has evolved from segregation towards integration and social inclusion. Alongside this shift, researchers have critically questioned what social inclusion means in practice for people with an ID, highlighting its multi‐dimensional and relational nature (Bigby 2012; Wiesel et al. 2024).

### 1.1 Social Inclusion

Social inclusion is a key determinant of health and wellbeing, particularly for people with an ID. The United Nations Convention on the Rights of Persons with Disabilities (CRPD: Rasmussen and Lewis 2007) explicitly recognises the right to “full and effective participation and inclusion in society” (Article 3), including “engagement in mainstream community services and groups, and emphasises participation in cultural activities, recreation, leisure, and sport” (Article 30). Despite these rights‐based commitments, many people with ID who wish to engage in their local communities remain “socially outside looking in” (p. 389, Gray et al. 2014). Experiences of stigma, discrimination, isolation, and loneliness persist, and opportunities for meaningful participation in inclusive community activities remain limited for many people (McCarron et al. 2013; Alexandra et al. 2018; Emerson et al. 2021).

A seminal used framework for conceptualising social inclusion for people with an ID is O'Brien and Lyle's (1987) clear distinction between “*community presence*” using facilities available to the general population, and “*community participation*” defined as belonging to a growing network of reciprocal relationships with people with and without disability. Verdugo et al. (2005) further emphasised that community participation and interpersonal relationships are core domains of quality of life for people with an ID. This distinction is particularly important when evaluating mainstream community‐based interventions, as presence alone does not necessarily translate into social integration and sustained inclusion.

Since O'Brien and Lyle's seminal work, conceptualisations of community participation and social inclusion have evolved considerably. Simplican et al. (2015) proposed an ecological model of social inclusion that conceptualises inclusion as the interaction between interpersonal relationships and community participation occurring within broader individual, organisational, community, and societal contexts. This framework highlights that social inclusion extends beyond attendance or presence in community settings and requires opportunities to develop meaningful relationships, reciprocal interactions, valued social roles, and a sense of belonging. More recent work has also emphasised the importance of everyday social interactions or convivial encounters within shared community spaces, where people with and without ID engage together in ordinary social activities. These encounters create opportunities for relationship development, mutual recognition, reciprocity, and sustainable community inclusion (Bigby et al. 2018; Wiesel et al. 2024).

Community participation is therefore increasingly understood as a relational and social process rather than simply physical access to community settings. Bigby et al. (2018) further argued that successful community participation programmes move beyond facilitating access to community locations and instead support meaningful social engagement, social connectedness, valued social roles, and enduring participation in mainstream community life. Such programmes often rely on relational supports, community capacity building, and mechanisms that foster inclusion within naturally occurring community networks.

### 1.2 Promoting Social Inclusion

Substantial efforts have been made to promote social inclusion by supporting people with an ID to engage in their local communities and develop broader social and interpersonal networks. However, the nature and intensity of support provided vary considerably across interventions and communities.

Several small empirical studies of 1–1 befriending interventions for adults with an ID (Florides 2012; Tse et al. 2021; Southby 2019; Ali et al. 2021; Brand et al. 2024), which, although promising, show limited effectiveness. Reported implementation barriers included recruitment and retention of befrienders/volunteers, difficulties in the matching process, transport and resource constraints, and variability in both participant and volunteer capacity and motivation. While 1–1 befriending interventions can facilitate access to community settings, they rely on individual‐level support and mainly offer a community presence, with limited opportunities for social inclusion and sustained engagement with community groups. In contrast, group‐based interventions that embed individuals with an ID within existing mainstream community groups offer greater potential for group‐level integration thereby offering more meaningful community participation, social inclusion and sustainable community integration (Giummarra et al. 2022; Killaspy et al. 2022; Stancliffe et al. 2023).

Stancliffe et al. (2015) developed the Transition to Retirement (TTR) community group‐based intervention, which supported older adults with an ID (< 45 years) who were leaving employment to join mainstream older person community groups. Using an Active Mentoring Model, retired community‐group members volunteered to act as mentors, supporting participants with an ID to engage in activities and social interactions at the group (i.e., bowling, gardening, crafts, Men's Shed). Participants attended groups weekly over six‐months, with qualitative findings indicating reduced loneliness, improved wellbeing, and expanded social networks. Notably, many participants continued attending the community groups after the intervention ended, suggesting sustainable community social inclusion and integration.

Bigby et al. (2014) undertook a further in‐depth explorative study to identify the conceptual components or programme logic of the TTR intervention so that it can be scaled and spread across other settings. There were three core components: “(1) promoting the concept of retirement, (2) laying the groundwork for inclusion of would‐be retirees with ID in the community, and (3) constructing the reality (planning, locating a group, mapping new routine, recruiting and training mentors, and monitoring and ongoing support).” (p. 117).

However, despite these promising outcomes of 1–1 befriending and community‐based interventions for adults and older adults with an ID, limited research has examined how such community‐based interventions are implemented and normalised within real‐world community settings, or the organisational and relational work required to sustain them. Importantly, many of these interventions, including the TTR model and related community inclusion initiatives, were researcher‐led or pilot programmes that were not subsequently adopted as routinely funded services (Craig and Bigby 2015a; Stancliffe et al. 2015). This raises wider questions concerning the translation of research evidence into policy, commissioning, and routine practice. While funding and policy support are critical determinants of long‐term sustainability, there remains limited understanding of the implementation work, relational processes, and organisational conditions required to successfully embed these interventions within real‐world community settings. Such knowledge is necessary to support both effective implementation and broader system‐level adoption.

### 1.3 The MATILDA Intervention

The MATILDA intervention (“Managing Activities Together to Involve older adults with a Learning Disability in their Local Areas”) was developed as a low‐cost, community‐based intervention adapted from the TTR model for the UK context (Taggart, Hassiotis, et al. 2026). MATILDA extends previous approaches by broadening eligibility beyond retirement transitions and placing explicit emphasis on group integration and sustainability.

MATILDA was designed as a structured, community‐based intervention that supported older adults with an ID (> 45 years) to become integrated within existing mainstream community groups (Taggart, Hassiotis, et al. 2026). Following recruitment to the intervention arm, participants met with the Volunteer Co‐ordinator (VC) who explored the participants' interests, hobbies, preferences, support needs, locality, age, and gender considerations. Suitable community groups were then identified and approached. Role of the paid staff have been to support (e.g., practical and emotional, transport, reassurance, etc.) the adults with ID to attend the community groups. Community group volunteers were recruited and trained to act as mentors, supporting the older adults with ID to attend the groups, develop relationships with other members, and become familiar with group activities and routines. The VC maintained regular contact with the adults with ID, mentors, carers, ID staff, and community organisations to facilitate problem‐solving, monitor progress, and support relationship development. As participants became established within their groups, support from the VC was gradually reduced with the intention that attendance and participation would be maintained through naturally occurring community relationships and existing community structures.

Given that the MATILDA feasibility study findings are reported elsewhere, only a brief summary is presented here to provide context for the current implementation analysis (see Taggart, Campbell, et al. 2026). The MATILDA feasibility randomised trial recruited 57 older adults with an ID across two UK sites (see Table 1 below). Findings demonstrated that recruitment, retention, mentor engagement, and community group participation were feasible. The adults with an ID reported positive experiences of community involvement, and most individuals allocated to the intervention continued attending community groups at six‐ and twelve‐month follow‐up.

**TABLE 1:** Characteristics of adults with intellectual disability participating in the MATILDA feasibility study.

Baseline characteristics	Treatment group		
	MATILDA	Control	Total
	*n* = 28	*n* = 29	*n* = 57
Male	13 (44.8%)	16 (55.2%)	29 (50.8%)
Female	15 (53.6%)	13 (46.4%)	28 (49.2%)
Age (years)	56.6 (7.1%)	56.7 (10)	56.68 (8.6)
Ethnicity: White	26 (92.9%)	23 (79.3%)	49 (86%)
Ethnicity: Black	1 (3.6%)	3 (10.3%)	4 (7.1%)
Ethnicity: Asian	1 (3.6%)	2 (6.9%)	3 (5.2%)
Ethnicity: Other	0	1 (3.4%)	1 (1.7%)
Living status: Supported living	13 (46.4%)	17 (58.6%)	30 (52.6%)
Living status: Own home	9 (32.1%)	6 (20.7%)	16 (28.1%)
Living status: Shared care	3 (10.7%)	0	3 (5.2%)
Living status: With Family	2 (7.1%)	1 (3.4%)	3 (5.2%)
Living status: Group home	1 (3.6%)	5 (17.2%)	6 (10.5%)

While these findings demonstrated preliminary evidence of acceptability, feasibility, and potential sustainability, they provided limited insight into the mechanisms and social processes that enabled MATILDA to become embedded within real‐world community settings. The present study therefore sought to address this evidence gap through an in‐depth implementation analysis using Normalisation Process Theory.

### 1.4 Normalisation Process Theory

Normalisation Process Theory (NPT) (May and Finch 2009; May et al. 2022) offers a robust theoretical framework for addressing this gap. NPT is a mid‐range sociological theory that focuses on the work that individuals and organisations collectively undertake to implement, embed, and sustain complex interventions (such as MATILDA) in real‐world settings. It conceptualises implementation as a dynamic social process involving four core constructs or social mechanisms: coherence or sense making (what is the work to be completed), cognitive participation or engagement (who does the work), collective action or enactment (how does the work get done), and reflexive monitoring or appraisal (how is the work understood/feedback) (Finch et al. 2013).

### 1.5 Aim and Objectives

This study used NPT to examine the social processes, mechanisms, and barriers that shape the implementation, embedding, and sustainability of the MATILDA intervention, designed to support community inclusion for older adults with an ID. Drawing on the perspectives of multiple stakeholder groups (adults with an ID, volunteers/mentors, community group managers, disability staff), this in‐depth qualitative study expands upon the results of the earlier MATILDA feasibility randomised trial (Taggart, Campbell, et al. 2026).

This study had four objectives, aligned with the core constructs of NPT:

1. To explore how the stakeholders made sense of the purpose and value of MATILDA (coherence or sense making).
2. To examine the engagement processes, mechanisms, and commitment among stakeholders involved in initiating and sustaining MATILDA (cognitive participation or engagement).
3. To identify how MATILDA was operationalised within mainstream community groups and disability services, including the work required to support participation (collective action or enactment).
4. To analyse how the different stakeholders appraised the effectiveness and barriers of MATILDA and its potential for long‐term sustainability (reflexive monitoring or appraisal).

While previous studies have demonstrated the benefits of community inclusion interventions and identified key intervention components that support participation (Bigby et al. 2014; Stancliffe et al. 2015), less attention has been paid to understanding how these interventions become embedded and sustained in routine community settings. In particular, there was limited understanding of the social, relational, and organisational work undertaken by the adults with ID, mentors, community‐group members, ID staff, and community organisations to support community inclusion in practice. The contribution of this study is therefore not to assess intervention effectiveness, but to provide a theoretically informed understanding of the implementation processes that support the normalisation and sustainability of community inclusion interventions.

## 2 Method

### 2.1 Design

Focus groups and 1–1 interviews were used to explore how MATILDA was implemented, embedded, and sustained within community settings, using NPT as the guiding analytical framework (May and Finch 2009; Murray et al. 2010). The study was designed to explore the social processes and mechanisms, and barriers, across multiple system or organisational levels.

### 2.2 Participants and Recruitment

Purposive sampling was employed to ensure representation across all stakeholder groups involved in MATILDA, capturing a range of perspectives, roles, and levels of engagement. This approach enabled exploration of social processes and mechanisms at individual, relational, and organisational levels. All MATILDA participants were given an opportunity to participate in the qualitative component of the study. Recruitment was facilitated by the ID community teams, who introduced the study and explained its aims and procedures to potential participants at each site. Accessible, user‐friendly participant information sheets and consent forms were used. Written informed consent was obtained by trained research staff before data collection.

Table 1 provides a summary of the characteristics of the sample including the participants' gender, age, ethnicity and living status split by both intervention and control arms. There were no differences between the adults with ID recruited from either the Northern Ireland or London sites.

In total, 29 individuals contributed qualitative data to this implementation study (see Table 2). In Northern Ireland, five focus groups and five 1–1 interviews were conducted. In London, one focus group and ten 1–1 interviews were conducted.

**TABLE 2:** Participants interviewed across stakeholder groups by site.

Site	Participants with ID	Mentors	ID service staff	Community group managers	Total interviewed
Northern Ireland	7	6	3	1	17
London	5	3	2	2	12
Total	**12**	**9**	**5**	**3**	**29**

### 2.3 Interview Schedule and Data Collection

Data were collected using semi‐structured focus groups and interviews informed by the NPT. Interview schedules were designed to explore the work involved in implementing and integrating MATILDA, structured around the four core NPT constructs: coherence or sense making, cognitive participation or engagement, collective action or enactment, and reflexive monitoring or appraisal. Separate interview schedules were developed for each stakeholder group, ensuring relevance to participants' roles while maintaining conceptual consistency across groups. Although theoretically informed, the semi‐structured format allowed flexibility to follow participants' priorities and experiences, facilitating in‐depth and reflective accounts of MATILDA's implementation.

Draft schedules were developed by the research team and reviewed by the wider trial management group. The interview schedule for adults with an ID was further reviewed and adapted in collaboration with an expert‐by‐experience group in Northern Ireland to enhance accessibility and comprehensibility.

To maximise comfort and accessibility, interviews and focus groups were conducted either in person (at community group venues, participants' homes, day centres) or online via Microsoft Teams, depending on participant preference. Reasonable adjustments were offered including additional time, use of familiar environments, the option to have a support person present, the use of visual aids to support communication and financial vouchers. All interviews were audio‐recorded with consent and lasted between 20 and 80 min.

### 2.4 Data Analysis

Audio recordings were transcribed verbatim, and analysis was informed by the principles of thematic analysis (Braun and Clarke 2022) but guided by a predefined deductive framework‐based approach informed by NPT (May and Finch 2009; May et al. 2022). NPT conceptualises implementation as four interrelated forms of work: coherence (sense‐making work), cognitive participation (engagement work), collective action (operational work), and reflexive monitoring (appraisal work). Each construct comprises four sub‐constructs that provide further explanatory detail regarding implementation processes and mechanisms. The analysis therefore focused on identifying data that illuminated the implementation, embedding, and sustainability of MATILDA across the four NPT domains of coherence, cognitive participation, collective action, and reflexive monitoring.

The analysis proceeded through five stages. First, transcripts were read repeatedly by members of the research team (AC, EK, ID) to achieve familiarisation with the data. Second, an initial coding framework was developed using the four NPT constructs and associated sub‐constructs. Third, transcripts were systematically coded using this framework, with data assigned to the NPT construct and sub‐construct that most closely reflected participants' experiences of implementing, supporting, participating in, and evaluating MATILDA. Fourth, patterns and similarities across stakeholder groups were explored and discussed during regular analytic meetings. Finally, interpretations were refined through investigator triangulation and review by the Chief Principal Investigator (LT) and London Principal Investigator (AH).

Rigour and trustworthiness were enhanced through use of a theoretically informed coding framework, investigator triangulation, transparent documentation of coding decisions, and regular team discussions to ensure consistency in the application and interpretation of the NPT constructs.

## 3 Findings

The findings were categorised into the four NPT constructs, which are defined as “the work that people do when they participate in implementation processes” (p. 3 May et al. 2022). Each construct comprises four sub‐constructs which provide further depth of the social processes and mechanisms, and barriers, for how MATILDA was implemented, embedded and integrated on both individual, collective, and organisational levels (see Table 3). To ensure anonymity of participants we have used abbreviations (NI = N Ireland; CI = Camden & Islington).

**TABLE 3:** Normalisation process theory concepts (taken from coding template based on May et al. (2022)).

NPT construct	Sub‐construct	Description
Coherence *How do people work together to understand and plan the activities that need to be accomplished to put the Matilda intervention and its components into place?*	Differentiation	*How do people distinguish the Matilda intervention and its components from their current ways of working?*
	Communal specification	*How do people collectively agree about the purpose of the Matilda intervention and its components?*
	Individual specification	*How do people individually understand what the Matilda intervention and its components required of them?*
	Internalisation	*How do people construct potential value of the Matilda intervention and its components for their work?*
Cognitive participation *How do people work together to create networks of participation and communities of practice around Matilda and its components?*	Initiation	*How do key individuals drive the Matilda intervention and its components forward?*
	Enrolment	*How do people join in the Matilda intervention and its components?*
	Legitimation	*How do people agree that the Matilda intervention and its components are the right thing to do and should be part of their work?*
	Activation	*How do people continue to support the Matilda intervention and its components?*
Collective action *How do people work together to enact the Matilda intervention and its components?*	Interactional workability	*How do people do the work required by the Matilda intervention and its components?*
	Relational integration	*How does using the Matilda intervention and its components affect the confidence that people have in each other?*
	Skill‐set workability	*How is the work of the Matilda intervention and its components appropriately allocated to people?*
	Contextual integration	*How is the work of the Matilda intervention and its components supported by host organisations?*
Reflexive monitoring *How do people work together to appraise the Matilda intervention and its components?*	Systemisation	*How do people access information about the effects of the intervention and its components?*
	Communal appraisal	*How do people collectively assess the Matilda intervention and its components as worthwhile?*
	Individual appraisal	*How do people individually assess the Matilda intervention and its components as worthwhile?*
	Reconfiguration	*How do people modify their work in response to their appraisal of the Matilda intervention and its components?*

NPT proposes that successful implementation and sustainability of complex interventions depend on four types of implementation work. Coherence refers to how stakeholders make sense of MATILDA and understand its purpose. Cognitive participation concerns the relational work required to recruit and sustain engagement from stakeholders. Collective action focuses on the practical work required to operationalise MATILDA within the community groups. Reflexive monitoring refers to how stakeholders evaluate and appraise MATILDA and its effects over time. Each construct is further divided into four sub‐constructs that help explain specific implementation processes and mechanisms (May et al. 2022). Table 2 summarises the NPT constructs and sub‐constructs that guided data analysis.

### 3.1 Coherence or Sense‐Making (What Is the Work To Be Done)

#### 3.1.1 Differentiation

Adults with an ID and mentors both readily distinguished that the MATILDA project was a process to enable older adults with an ID to join existing mainstream community groups. For the adults with ID, MATILDA enabled opportunities for new social connections and skill development, contrasting with feelings of exclusion in other contexts. This may reflect a strong sense of community inclusion, with participants feeling no marked difference between themselves and others in the groups. One adult with an ID stated *“*…*it was more like interacting with other* [non‐disability] *people, and we* [adults with ID] *didn't feel an outcast…”* (CI/Participant 2).

The mentors and community managers emphasised how MATILDA provided a bridging role, supporting the smooth transition of adults with an ID into the groups. One manager from an ID setting explained “…it's about social inclusion, to be able to get people with a disability to access community facilities…rather than being excluded because they have got a learning disability” (NI/ID Manager 2). In contrast, some of the ID staff reported a limited understanding of the purpose and value of the MATILDA project due to high staff turnover and communication breakdowns, seeing MATILDA as no different to other ID groups. This lack of clarity restricted a few of the adults with an ID from attending their community groups.

#### 3.1.2 Communal Specification

There was a shared understanding from all stakeholders that MATILDA was clearly successful in matching and sustaining many of the older adults with an ID to mainstream community‐based groups and mentors. The core role of the two VCs was viewed as instrumental in facilitating this matching process by building rapport and trust between the adults with an ID and their mentors. This also included regular communication with the family carers and ID staff to ensure smooth transition into the group and diminishing any fear they may hold. The VC's individual role also enabled the dyad to become less reliant on the support offered by them and eventually for the VC to withdraw completely within six months or less from this supportive role.

#### 3.1.3 Individual Specification

The adults with ID initially had a limited understanding of their role other than in terms of attendance at the groups with their mentors. However, as the participants developed their sense of belonging and confidence, this widened their friendships with the other members of the group, quickly learning their tasks and responsibilities, which were welcomed by other group members.

Mentors reported greater role clarity, describing their specific responsibilities from welcoming the adult with an ID into the group, building rapport, and easing transitions. One mentor highlighted the importance of a “whole community group approach”, suggesting that the responsibility of supporting and integrating the adults with an ID into each group should be shared among the wider group members, highlighting the importance of *“group buy‐in”*. The mentor's role was often viewed as supportive but not intensive, as one mentor described:

I do very little, I stand back and I let the people [other members of the community group] then create the atmosphere…but my role is to make sure that there is a positive atmosphere without getting too heavy handed with people [participant with the ID]. (NI/Mentor 5)

Community managers described their role as the “*middle person” (CI/Community Manager 2)*, working alongside the VC to identify suitable groups and provide support to mentors. The ID staff described both supportive functions such as information sharing and safeguarding but also noted challenges, including sustaining participation, arranging support, providing transport and managing risks. For those adults with an ID attending without paid staff, faced further challenges in participating in some group activities. These accounts suggest that while role clarity was strong for the VCs and mentors, it was more fragmented among the ID staff.

#### 3.1.4 Internalisation

The adults with an ID clearly articulated the personal and social benefits they gained from MATILDA including increased confidence, reduced loneliness, meaningful activity engagement, and improved quality of life. Mentors highlighted MATILDA's role in *“fostering inclusion”*, as well as *“opportunities for their own learning and leadership development”*. Community managers emphasised broader community‐level benefits, including *“raising awareness of disability and promoting diversity within the community groups.”* One community manager noted *“they* [mentors and other group members] *learn more about how they engage with people with a learning disability…this has been a new experience for them…”* (CI/Community Manager 2). Further, one ID manager stated MATILDA acts as *“an enabler of social inclusion*” and another member of staff similarly affirmed *“so it's just integration isn't it”* (CI/ID staff 2).

### 3.2 Cognitive Participation or Engagement (Who Does the Work)

#### 3.2.1 Initiation

The VCs were the principal drivers of MATILDA implementation. They worked closely with the ID services, community‐group managers, mentors, family carers, and adults with ID to establish participation pathways, identifying suitable community groups, facilitating mentor recruitment, and developing relationships across stakeholder groups. Community ID teams supported this process by identifying potential older adults with ID and facilitating engagement. The VCs' ongoing leadership and co‐ordination were widely viewed as essential to initiating and maintaining involvement within community groups.

ID staff reinforced initiation through easy‐read information and ensuring routines were predictable: *“he needs to know all these things… we just need to make it happen”* (NI/ID Manager1). At the same time, initiation was constrained by staff shortages, transport, mobility limitations, and reliance on rota management. ID staff and managers also raised the issue of having to ensure a particular gender of staff was required for specific gender community groups (i.e., Men's Shed, women only groups) posing challenges that further restricted implementation.

#### 3.2.2 Enrolment

For the adults with an ID, enrolment often required overcoming initial nervousness. One participant reflected, *“first, I got really nervous… after time you get used to everyone in the group and what to do”* (CI/02). However, for some participants with an ID, engagement was usually contingent on the VCs' motivation and support: *“I would love to go… as long as I have the lady* [VC], *I need her to support me”* (CI/04).

Mentors facilitated enrolment by positioning the adults with an ID as ordinary members: *“I never said to anybody that X is this or that… they just took him to their hearts”* (NI/Mentor 05). Group members supported enrolment by offering inclusive and varied activities (e.g., knitting, art groups, drop‐ins), which sometimes provided stepping stones into broader social opportunities: *“Knit and Knatter led to afternoon teas at the social club and other excursions”* (NI/Mentor 03).

#### 3.2.3 Legitimation

All the stakeholder groups widely agreed that MATILDA was a valuable and appropriate community‐based intervention for the older adults with an ID, though belonging was not always straightforward. One man with an ID described uncertainty: *“I did feel part of the group, but sometimes I felt like I didn't and I did”* (CI/02).

Mentors legitimatised MATILDA by emphasising the value of inclusion outside disability‐only contexts: *“how great to hear that he's chatting to non‐disabled people… it's absolutely invaluable”* (CI/Mentor 02). ID staff recognised the quality‐of‐life benefits *“they spend a lot of time here* [residential facility] *… some just stay in their room and they're missing out*” (CI/ID staff 02). Community managers legitimatised MATILDA by aligning it with the groups' organisational values and practices. For example, one manager reported that their church group has *“updated their policies to align with MATILDA's ethos of community inclusion for all people with a disability”* (NI/Community Manager 01).

ID staff highlighted MATILDA's importance for offering social opportunities *“it's promoting a sense of belonging… instead of everything being medicalised”* (NI/ID staff 03). Despite strong support, ID staff acknowledged that MATILDA was not suitable for all, noting the importance of identifying the *“right person”* (CI/ID staff 02) for the right group to ensure smooth participation and provide a valid contribution to the group.

#### 3.2.4 Activation

Sustaining motivation, integration, and engagement over the six and twelve months of MATILDA was contingent on each group's adaptation and continuity. The adults with an ID valued consistency *“if the mentor is not there all the time, how can I get the mentor?”* (CI/02). Rapport, trust, and communication with the VCs, the mentors, and other group members reinforced ongoing involvement and sustainability.

Community groups sustained participation by embedding the adults with an ID as equal members while avoiding overprotection *“we don't treat him any different… we're mindful, but we don't wrap them in cotton wool”* (NI/Mentor 02). Activities were also adapted to accommodate different abilities *“we mix and match… so if* X *is weaker, we put stronger people on the team”* (NI/Mentor 04). Nonetheless, sustainability remained fragile with those participants living in ID services, often undermined by staffing gaps (i.e., numbers of staff on duty, competing demands, gender of staff, transport) and rota inflexibility.

### 3.3 Collective Action or Enactment (How Does the Work Get Done)

#### 3.3.1 Interactional Workability

The adults with an ID described the adaptations that supported inclusion. Flexibility extended beyond single sessions, when an activity failed, mentors sought alternative pathways *“we still want to get* [participant] *involved. And we thought… we've got a Lego Club”* (CI/Mentor 01). For a few participants with an ID, sensory sensitivities limited their ability to interact in group environments *“there was one person what was really loud… I was getting wound up… so I just listened to my music… went into my own little world”* (CI/02). Crucially, interaction was often embedded in shared activities, with the adults with ID emphasising the enjoyment of *“just sit and talk and chat and just knit”* (NI/03). These findings demonstrated that the practical workability of MATILDA depended on balancing structured activities with opportunities for informal social interaction, supported by the presence of VCs, mentors and other group members.

#### 3.3.2 Relational Integration

A central theme was the interpersonal work of the VC at each site, at each group, who reinforced engagement through reminders and check‐ins. Others valued the VC's attentiveness *“it's nice of* [VC] *calling in when he was in X to see how we were getting on”* (NI/03).

Mentors were instrumental in building trust, confidence, and providing tailored practical and emotional support. One adult with an ID explained *“I can't read and I can't write… and* [mentor] *helped me with that kind of thing and that really helped me”* (CI/02). Peer friendships developed within groups and were similarly valued, *“they* [group members] *are kind and friendly”* (CI/Participant 05). Community managers noted that the rapport between the VC and mentors inspired confidence in the adults with ID *“you felt confident that the VC and mentor had thought of everything”* (NI/Community Group Manager 01). Community group members also recognised the role of the VC in fostering a sense of belonging *“the woman* [VC] *in charge is really nice… she really relates to* [participant name]. *All those little things make a massive difference”* (CI/Community Group Manager 01).

Despite these advantages, reliance on the VC occasionally created relational vulnerability. As one group member observed, *“being reliant on having the VC there*… *and then gradually weaning off”* (CI/Community Group Manager 02). The need for a gradual transition away from the VC highlighted the relational intensity of embedding new practices and the potential risks if that support is withdrawn too abruptly.

#### 3.3.3 Skill‐Set Workability

Matching the adults with an ID with a community group and mentors was based on shared interests/hobbies/activities and was identified as key to success. Mentors described confidence in their roles, offering practical strategies for inclusion *“Get to know ’em personally, perhaps call them by their first name. Always greet them, always open a door for them and understand their needs”* (CI/Mentor 03).

Training offered by the VCs was highly valued, both as reassurance for those new to working with people with an ID and as a source of fresh insights for experienced staff. One mentor stated, *“I would go back to the notes that the VC gave me… it was just really helpful to know that I had these notes that I could go back to”* (CI/M 01). Another noted, *“even though I do work with people with learning disability… I certainly learned there's information about communication and some good pointers… that's really helpful”* (CI/Mentor 02).

However, some mentors identified gaps in preparation, particularly around risk management. *“I just think embedding risk assessments is important… I'm really risk averse”* (CI/Mentor 02). In contrast, some groups perceived formal training as unnecessary or undesirable. *“our boys* (other group members) *would run a mile at the thought of formal training”* (NI/Mentor 02). More broadly, some community groups reported that the ethos of inclusion was already embedded in their practices. *“our members are generally really supportive and so it wasn't anything that they had to adapt”* (CI/Community Group Manager 02). These findings suggest that while targeted training added value, MATILDA was most sustainable where it aligned with existing values and skillsets within community groups.

#### 3.3.4 Contextual Integration

Transport consistently emerged as a critical barrier. Some adults with an ID reported both logistical and financial constraints *“it cost me £20 to go to the Men's Shed”* (NI/04). ID staff also described challenges in arranging suitable transport and rotas *“making sure… that I have the staff in place that have the transport organised… and that we were able to get a time that suited both them and us”* (NI/ID staff 02). At times, transport unreliability undermined participation *“we booked the cab the day before… and the cab never came”* (CI/ID staff 01).

### 3.4 Reflexive Monitoring or Appraisal (Evaluating the Work)

#### 3.4.1 Systemisation

Stakeholders described a range of formal and informal mechanisms through which information about MATILDA participation and progress was gathered and shared. Regular contact with the VCs, ongoing communication between community groups, mentors, ID staff and family carers, and reporting arrangements enabled stakeholders to monitor attendance, address emerging issues, and evaluate participants' integration into community groups. Community‐group managers particularly valued the communication processes established by the VCs, describing reporting mechanisms as effective and responsive. These feedback processes enabled stakeholders to understand the impact of MATILDA and make reasonable adjustments where required.

Reporting mechanisms with the VC were described as effective: *“reporting mechanisms were good. We were happy with that… and just how we were communicated with”* (NI/Community Group Manager 01). However, where trained mentors were not consistently available, participation was compromised: *“now that she is moved away from the initial class, she's no longer with her designated mentors”* (CI/Community Group Manager 02).

#### 3.4.2 Communal Appraisal

Appraisal was strong across all stakeholder groups, especially around the value of the support of the VCs, mentors and wider group members. The adults with ID described that they liked participating in the group's different social activities and external activities. Across all the stakeholder groups, the participants consistently reported a range of benefits including increased confidence, learning new skills, developing friendships, a stronger sense of belonging, improved self‐worth, and enhanced emotional wellbeing. These perceptions were expressed by adults with ID, mentors, ID staff, and community group managers: *“he's much more confident now and talks to people he would never have spoken to before”* (NI/Mentor 05) and *“I've made new friends here and I enjoy coming every week”* (NI/Participant 07). Group enjoyment and the social atmosphere were framed as a shared experience for all rather than individual benefit alone.

Mentors and community managers also provided collective judgements. Some managers described MATILDA as *“a success” and something they wanted to buy into and build on,”* especially as a *“first”* for their organisation. Mentors highlighted that groups themselves did not have to fundamentally change; the aim was to find a good fit between an existing group and the person with ID, then match to one or more mentors, rather than redesign the group around MATILDA. Communal appraisal also captured shared recognition of challenges: ensuring participants were safe and well when they did not show up, dealing with gender imbalance (e.g., a man with a ID reluctant to attend a group of all women), and acknowledging that not every match would work despite everyone's best efforts.

#### 3.4.3 Individual Appraisal

The adults with an ID described MATILDA as personally rewarding, highlighting increased confidence, enjoyment, independence, and opportunities to form meaningful friendships. Several participants with ID reflected on how attending community groups had become an important part of their weekly routine and contributed positively to their sense of identity and belonging. They also described clear personal and social benefits of MATILDA “discovering new activities (art classes, gardening, outings, coffee mornings), joining additional groups” (NI/P06). However, participants with ID also reported that trips were “enjoyable but sometimes short, transport and travel time were burdensome, groups were often booked up, and some people changed their mind at the last minute or felt groups were “not their cup of tea” (NI/P03). This showed the person with ID actively weighing effort, access, and enjoyment when judging impact.

ID staff and managers systematically reflected on broader outcomes highlighting “increased friendships, confidence, independence (e.g., using transport, doing things beyond the day centre), and better mental health when people were “out among people” rather than at home” (NI/ID staff 07).

Mentors similarly reported personal satisfaction from their involvement, describing the role as rewarding and meaningful. Many reflected on the value of witnessing positive changes in the adults with ID, confidence, social engagement, and willingness to try new activities. Community group managers also appraised MATILDA positively, recognising its contribution to community inclusion and wellbeing while acknowledging the effort required to support participation. These personal evaluations reinforced stakeholders' commitment to sustaining community involvement beyond the formal intervention period.

#### 3.4.4 Reconfiguration

The adults with ID offered concrete ideas for redesign: widening MATILDA to more younger and diverse disability groups but ensuring age‐matched groups and more personalised matching based on hobbies and preferences. They also wanted more frequent check‐ins from the research team (e.g., phone calls after groups) and longer or more sustained contact with the VC. Some adults with ID changed their own behaviour in response to their positive appraisal, seeking additional groups online and planning to continue exploring community activities beyond MATILDA.

Mentors reported modifying their own practice, shifting from a *“doing for”* approach to actively empowering adults with ID to take the lead. ID staff described reconfiguration at the service level. MATILDA had *“helped them access parts of the community they didn't know existed”* (CI/ID staff 01) and prompted them to seek more community activities for other adults with ID. ID staff spoke about being encouraged and challenged to *“start seeking new things”* (CI/ID staff 01), indicating a shift in the routine practice of community inclusion. Community group managers also began to adapt. They recognised the need for clearer processes when the adults with ID did not turn up, and for more awareness about disability among the public, suggesting that future work could include preparatory information or training for wider group members.

## 4 Discussion

This study extends MATILDA (Taggart, Campbell, et al. 2026) and previous community‐based research (Bigby et al. 2014; Stancliffe et al. 2015) by moving beyond questions of intervention effectiveness, feasibility, and programme design to examine the implementation processes that underpin successful community inclusion. Applying the NPT has enabled exploration of how multiple stakeholders collectively understood, initiated, enacted, supported, and evaluated MATILDA, thereby providing implementation insights that can inform future scale‐up and sustainability of community inclusion interventions.

### 4.1 Coherence or Sense‐Making (What Is the Work To Be Done)

Across the different stakeholder groups, the MATILDA project was generally understood as novel and distinct offering support for the adults with an ID to engage in mainstream older people's community groups. The VCs, mentors, and wider community group members understood their specific tasks and responsibilities. Likewise, the ID staff recognised their role in supporting the participants to attend the community groups, although they did voice some logistical challenges. These social processes and mechanisms of shared understanding and sense‐making of MATILDA's purpose and value helped successfully initiate, embed, and sustain the adults with an ID to remain within their community groups long after the VCs withdrew at six‐months and were still attending at twelve‐months (Taggart, Campbell, et al. 2026).

### 4.2 Cognitive Participation or Engagement (Who Does the Work)

There was strong evidence of cognitive participation or engagement from the VCs, mentors, wider group members, and ID staff reflecting in their commitment to working together in supporting the adults with an ID to participate meaningfully in the community‐based groups. This cohesiveness further reflected the different stakeholders' values around social inclusion, reciprocity, and community connection. These social processes and mechanisms focused upon the interpersonal relationships that developed between the VCs, mentors, wider group members, and adults with ID through clear communication, trust, and equal encounters. These social processes and mechanisms were echoed by colleagues who explored the workings of the TTR in Australia (Bigby et al. 2014; Stancliffe et al. 2015).

### 4.3 Collective Action or Enactment (How Does the Work Get Done)

Our results confirm the findings of the study by Wiesel et al. (2024) that explored the factors that supported and integrated people with an ID into mainstream community services in Australia. The authors used a relational approach to explore the interrelationships between the adults with ID, mainstream community, and ID staff. Wiesel and colleagues identified seven key characteristics that showed positive interpersonal relations and interactions. These were: *‘respect; warm, welcoming and convivial interpersonal engagement; active listening; proactive assistance; flexibility; mediation to create a safe environment; and collaboration and shared responsibility by both mainstream staff and ID support workers in supporting a service user with ID’* (p. 391). Like our study, Wiesel et al. (2024) concluded that a *“web of relations and complexity”* is involved in supporting and integrating people with an ID into mainstream community‐based services.

Participants across all our stakeholder groups described how the older adults with an ID were further supported and integrated within the community groups. Some of these early social processes and mechanisms included careful matching of individuals to groups and mentors based on shared interests, hobbies, and activities. This group‐level flexibility to make reasonable adjustments, balanced between group activities and informal social interaction, was clearly valued by the adults with ID. These adaptive practices enabled the participants to be integrated into existing community‐based group routines without disrupting the group's core purpose, supporting its operational fit and workability. Further social processes and mechanisms of the MATILDA project focused on community‐group adaptability, flexible adjustments and shared responsibility, facilitating inclusive participation while maintaining the integrity of the community group.

### 4.4 Reflexive Monitoring or Appraisal (Evaluating the Work)

Mentors, community group managers, and ID staff consistently evaluated and appraised the positive impacts associated with MATILDA, including the increase of new friendship networks, self‐confidence and greater sense of belonging, reduced social isolation, improved wellbeing, and enhanced community inclusion for the participants with ID. These accounts were corroborated by the adults with an ID themselves, who articulated the personal and social benefits and improvements in quality of life of engaging in the community groups. These findings of MATILDA have been further validated by Taggart, Campbell, et al. (2026) and similarities found within the previous TTR project (Craig and Bigby 2015a; Stancliffe et al. 2015).

### 4.5 Core Mechanisms and Social Processes Underpinning MATILDA

A core mechanism underpinning MATILDA was the appointment and active role of the VC at each site in Northern Ireland and London. The VC functioned as a key agent of implementation by facilitating coherence, enabling collective action, and supporting sustained engagement across stakeholders. Earlier 1–1 befriending interventions (Florides 2012; Southby 2019; Tse et al. 2021; Ali et al. 2021; Brand et al. 2024) and community‐group‐based interventions (Craig and Bigby 2015a; Bigby et al. 2018; Stancliffe et al. 2015) also employed VCs and identified their key role as implementation champions.

The mentor role also emerged as another core mechanism supporting MATILDA's success. Mentors were central to the day‐to‐day enactment of supporting and integrating the adult with an ID into the community groups. Their work included welcoming participants into the group, building rapport, easing transitions into new social environments, and safeguarding both individual wellbeing and group dynamics. Mentors also adapted activities where appropriate to support participation while maintaining the integrity and routine of the group. Wilson et al. (2013) found that using an Active Mentoring Model in the TTR project, mentors who were offered some training could successfully support a person with an ID to be included within a local community group.

MATILDA's implementation was further supported by wider social processes as the adults with ID contributed to their community group through their motivation, growing capability, and willingness to adapt to community group contexts. The wider community groups' members played a critical role through a *“whole‐group approach”* characterised by *“group‐level buy‐in”*, shared responsibility for inclusion, and the interaction of adults with an ID as equal members. Community group managers acted as *“middle agents”*, working alongside VCs and mentors to align MATILDA with existing group structures, routines, and safeguarding requirements.

Together, these social processes and mechanisms demonstrated that all the stakeholders functioned collectively to successfully make MATILDA work. As articulated by one community group manager, MATILDA operated as a *“bridge”* between ID services and mainstream community‐based groups. While prior research has examined the mechanisms underpinning befriending (Ali et al. 2021; Cavender 2022; Robinson and Idle 2023; Brand et al. 2024) and community‐based interventions (Craig and Bigby 2015a; Stancliffe et al. 2015; Bigby et al. 2018), these studies have tended to focus on programme components or logic. In contrast, this study makes a novel contribution by explicating the relational, organisational, and interactional work undertaken by multiple stakeholder groups to embed MATILDA within mainstream community‐based groups.

### 4.6 Relational Work as a Core Implementation Mechanism

While many practical features of MATILDA such as participant matching, mentor recruitment, community group identification, and ongoing support have been described previously within the TTR literature (Bigby et al. 2014; Stancliffe et al. 2015), our findings suggest that these structural components alone do not explain successful implementation and sustainability. Rather, the success of MATILDA appeared to depend on the relational work occurring between stakeholders. Across all four NPT constructs, participants described the importance of trust, rapport, reciprocity, communication, responsiveness, flexibility, and shared responsibility. VCs acted as relational brokers, creating connections between ID services and community groups, while mentors facilitated belonging through everyday interpersonal interactions rather than formal support alone.

Equally important was the role of wider community group members, whose acceptance of the adults with ID as ordinary participants fostered a sense of inclusion and membership. These findings extend previous descriptions of the programme logic reported within the previous TTR project (Bigby et al. 2014; Stancliffe et al. 2015) by demonstrating how implementation was achieved through a network of social relationships rather than through the intervention components alone. In this sense, MATILDA functioned not simply as a community inclusion programme but as a relational intervention, where successful implementation depended on building and sustaining interpersonal connections across community, ID, and volunteer contexts.

### 4.7 Barriers to Implementation

ID staff supported the implementation and sustainability of MATILDA by identifying suitable participants, providing contextual information, offering practical support (such as transport and accompaniment), and managing risk, alongside emotional support for participants. However, coherence varied with some ID staff demonstrating limited understanding of MATILDA's aims and how it differed from existing disability groups. Initiation, enactment, and sustainment of MATILDA were constrained by staff shortages, transport, mobility limitations, and reliance on rota management. These findings emphasise the importance of identifying and addressing organisational system level factors and highlight how systemic pressures can undermine the normalisation of otherwise acceptable and effective interventions. Similar findings have recently been reported by Kelly et al. (2025) who found the greatest challenge in an ID randomised trial was not the participants with ID, but the organisational system level factors such as *“getting senior and front‐line staff buy‐in and engagement, and discomfort of staff putting people with ID forward”* (p. 1).

More broadly, the sustainability of community inclusion interventions may depend not only on successful implementation within community settings but also on policy, commissioning, and funding decisions. Previous community‐based programmes, including the TTR project, demonstrated positive outcomes yet were not universally adopted as funding was not provided long‐term (Craig and Bigby 2015a; Stancliffe et al. 2015). Consequently, implementation knowledge alone is unlikely to guarantee spread and sustainability. Rather, understanding implementation processes through frameworks such as NPT should be viewed as complementary to efforts aimed at strengthening policy uptake, service commissioning, and the translation of evidence into routine provision.

### 4.8 Strengths and Shortcomings

This study's strength lies in its use of NPT alongside the perspectives of older adults with ID, mentors, community managers, and ID staff, providing a rich and theoretically informed analysis of implementation processes in real‐world settings. A particular strength of the paper is that it moves beyond intervention outcomes to examine the relational and organisational work required to embed and sustain community inclusion interventions in routine practice.

However, a potential limitation of this study was its use of a predefined NPT‐informed deductive analytic framework. While this enabled systematic examination of implementation processes across stakeholder groups, this approach differs from reflexive thematic analysis (Braun and Clarke 2022) and may have constrained the emergence of themes outside the predefined framework. An inductive analysis may have yielded additional insights regarding participants' lived experiences of community inclusion. Future research could usefully combine inductive thematic approaches with implementation frameworks to capture both experiential and implementation dimensions of interventions such as MATILDA.

Other implementation science frameworks such as the Consolidated Framework for Implementation Research (CFIR 2.0: Damschroder et al. 2022) could have been used to explore further the broader organisational determinants (inner and outer settings, participant characteristics, innovation or intervention features, and implementation processes) for MATILDA. Future research may benefit from theoretically integrated approaches that combine both NPT and CFIR to further unpack how context and organisational system level factors shape the normalisation of inclusive community‐based interventions in real‐world settings.

## 5 Conclusion

A key contribution of this study is its identification of the relational mechanisms, including trust, reciprocity, communication, and shared responsibility, that underpin MATILDA's successful implementation and long‐term community inclusion. This novel approach has shown how MATILDA has been operationalised, normalised, and sustained within real‐world settings. Applying the NPT has enabled a nuanced exploration of how MATILDA was understood, initiated, engaged, enacted, and appraised by multiple stakeholder groups. By leveraging existing community infrastructure and volunteer capacity, MATILDA has the potential to provide a low‐cost, sustainable intervention that reduces loneliness and mental ill‐health for adults with an ID while decreasing reliance on formal health and social care services.

## Author Contributions

L.T. Lead PI, obtained the funding, wrote 1st draft of paper. A.C. supported Lead PI in N Ireland, obtained ethics, conducted the focus groups, analysed the data, supported with paper writing. I.D. supported co‐PI in London, obtained ethics, collected the data. A.H. Co‐PI in London; supervised research staff at site; helped write the paper. K.E. supported co‐PI in London; was the Volunteer Co‐ordinator in London; helped conduct the focus groups; helped draft the paper. H.A. supported co‐PI in London, obtained ethics, collected the data. A.H. supported Lead‐PI in N Ireland; was the Volunteer Co‐ordinator in N Ireland; helped conduct the focus groups; helped draft the paper. B.B. Co‐PI, trial statistician; helped draft the paper. C.O. Co‐PI, health economist; helped draft the paper. M.C. Co‐PI, trial methodologist, helped draft the paper. L.A. co‐PI, supported Volunteer Co‐ordinator in N Ireland; helped draft the paper. B.L. co‐PI, supported Volunteer Co‐ordinator in London; helped draft the paper. J.S. co‐PI; PPI lead; helped draft the paper. S.W. supported Volunteer Co‐ordinator in London; helped draft the paper. A.R. co‐PI, supported methodological design of study, helped with data analysis, helped draft the paper. R.S. co‐PI, supported methodological design of study, helped draft the paper.

## Funding

This study was funded by the National Institute for Health and Care Research (NIHR) Public Health Research (PHR) Programme (NIHR129125). The views expressed are those of the authors and not necessarily those of the NIHR or the Department of Health and Social Care, UK.

## Ethics Statement

Ethical approval was obtained from the Office for Research Ethics Committees Northern Ireland (ORECNI: 22/NI/0067), and research governance approvals were secured at both study sites. Audio recordings and transcripts were stored securely on password‐protected systems, anonymised during transcription, and managed in accordance with GDPR requirements and local data protection policies.

## Consent

Consent has been obtained from all authors to publish this paper.

## Conflicts of Interest

The authors declare no conflicts of interest.

## Data Availability

A full anonymised transcription of the interviews coded to the four constructs of the Normalisation Process Theory is available on request from the lead author on reasonable request.
